# A recurrent retrohepatic abscess secondary to a dropped appendicolith

**DOI:** 10.1016/j.radcr.2023.12.040

**Published:** 2024-01-04

**Authors:** Shahzeb Sheikh, Muskan Kaur, Abed M. Zaitoun, Dileep N. Lobo

**Affiliations:** a Nottingham Digestive Diseases Centre, Division of Translational Medical Sciences, School of Medicine, University of Nottingham, Nottingham NG7 2UH, UK; b National Institute for Health Research (NIHR) Nottingham Biomedical Research Centre, Nottingham University Hospitals NHS Trust and University of Nottingham, Queen's Medical Centre, Nottingham NG7 2UH, UK; c MRC Versus Arthritis Centre for Musculoskeletal Ageing Research, School of Life Sciences, University of Nottingham, Queen's Medical Centre, Nottingham NG7 2UH, UK

**Keywords:** Appendicolith, Complications, Perforated appendicitis, Perihepatic abscess, Treatment

## Abstract

Appendicoliths can drop into the peritoneal cavity during the course of an appendicectomy, or more commonly as a result of perforated appendicitis. We report the case of a patient with a history of recurrent retrohepatic abscesses over 7-year period due to a retained appendicolith and review the literature on perihepatic abscesses caused by retained appendicoliths. The abscess had been drained percutaneously 4 times without retrieval of the appendicolith and eventually the patient needed a laparotomy, drainage of the abscess, and extraction of the appendicolith. Treatment of abscesses secondary to dropped appendicoliths may be percutaneous, laparoscopic, or via conventional open surgery, but it is important to retrieve the appendicolith if recurrent abscess formation is to be avoided.

## Background

Appendicoliths are formed within the lumen of the vermiform appendix due to aggregation of faecal matter and inorganic salts, and usually present as calcific masses within the appendicular lumen [1,2]. Appendicoliths may be identified on computed tomography (CT) scanning in up to 30% of individuals [3] and although they do not predispose to appendicitis *per se*, their presence is a risk factor for failure of nonoperative treatment of acute appendicitis [4]. Appendicoliths can drop into the peritoneal cavity during the course of an appendicectomy, or more commonly as a result of perforated appendicitis. Retained or dropped appendicoliths can migrate to various parts of the peritoneal cavity, retroperitoneum, or even the chest, and result in abscess formation [1,^2^,[5], [6], [7], [8], [9].

We report the case of a patient with a history of recurrent retrohepatic abscesses due to a retained appendicolith and review the literature on perihepatic abscesses caused by retained appendicoliths.

## Case history

**Fig. 1:**
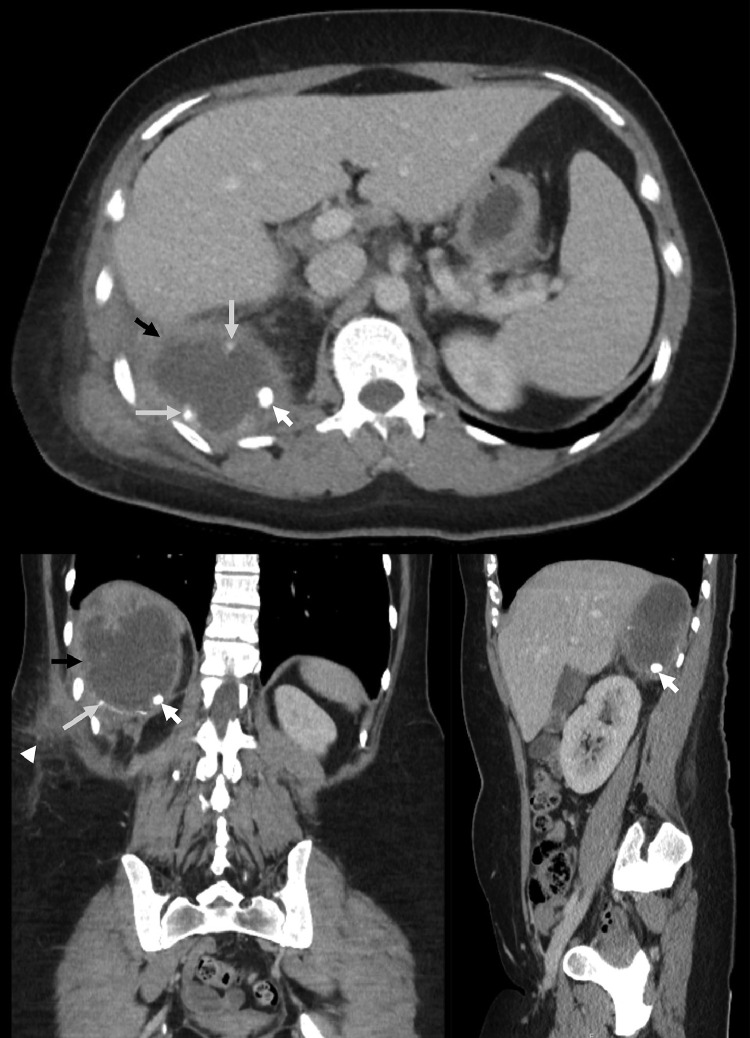
Axial (top), coronal (bottom left) and sagittal (bottom right) CT images showing a 9 cm retro hepatic abscess (black arrows) with a 10 mm appendicolith (white arrows) and an extension into the subcutaneous and intramuscular compartments (white arrowhead). Specks of calcification were also noted in the wall of the abscess cavity (gray arrows).

**Fig. 2:**
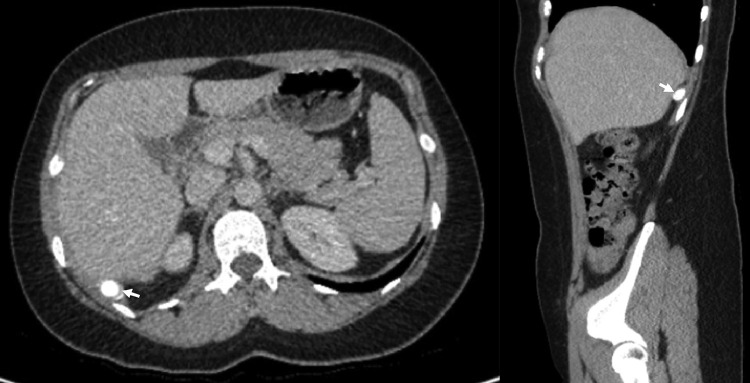
Axial (left) and sagittal (right) interval CT images obtained 4 years previously showing a 10 mm retrohepatic calcified body (white arrow).

A 37-year-old woman who had a laparoscopic appendicectomy 7 years previously for perforated appendicitis was referred to a tertiary center with a 3-day history of right-sided upper abdominal pain radiating to the back. There was no fever, nausea or vomiting. General physical examination was unremarkable, but there was erythema and tenderness posteriorly over the lower ribs on the right side. There was no history of a cholecystectomy or acute cholecystitis. The white cell count and C-reactive protein concentration were elevated at 14.1 × 10^9^ cells/L (normal range 4-11 × 10^9^ cells/L) and 193 mg/L (normal range 0-10 mg/L) respectively. Since the appendicectomy, she was diagnosed with a right-sided retrohepatic abscess that had been drained percutaneously under transabdominal ultrasound guidance at the referring center on 4 occasions, the first being 3 weeks after the appendicectomy and the last 4 months previous to the present admission. There was no drain *in situ* at the time of the most recent presentation. An abdominal CT scan on this occasion showed a thick-walled collection measuring 94 × 65 mm posterior to the right lobe of the liver. There was a calcified body measuring 10 mm in diameter within the abscess cavity. There was also a thick-walled collection measuring 60 × 29 mm mostly within the subcutaneous and intramuscular compartments of the right posterior thoracoabdominal wall (Fig. 1). Specks of calcification were also noted in the wall of the abscess. There was no intrahepatic pathology, and the rest of the abdominal viscera were normal. An interval CT scan done 4 years previously also showed a 10 mm retrohepatic calcified body (Fig. 2), which was also noted on the first CT scan done 3 weeks after the appendicectomy.

**Fig. 3:**
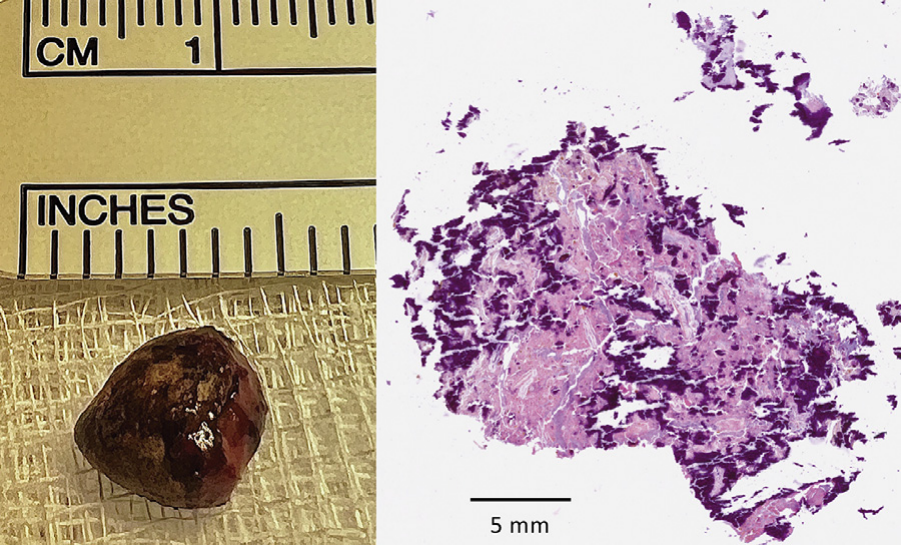
Gross (left) and microscopic image (right) of the appendicolith retrieved at surgery. Microscopy showed fibrinous material, degenerate cells and calcification. There was no evidence of mucin or malignancy.

She underwent a laparotomy via a right subcostal incision and was found to have a chronic abscess cavity approximately 10 cm in diameter posterior to the right lobe of the liver. The right lobe of the liver was mobilized, the abscess was drained and the appendicolith (Fig. 3) was retrieved. Lavage of the abscess cavity was performed with 0.9% saline and a drain was left *in situ*. Culture of the pus revealed the growth of *Escherichia coli* sensitive to co-amoxiclav, gentamicin, ciprofloxacin, and piptazobactim but resistant to amoxicillin and penicillin. She was treated with a course of co-amoxiclav and made an uneventful recovery and was discharged on the 5th postoperative day after removal of the drain. Histological examination revealed fibrinous material, degenerate cells, and calcification within the appendicolith (Fig. 3). There was no evidence of recurrent abscess formation when last seen 6 months postoperatively.

## Discussion

The present case highlights the importance of making an effort to search for dropped appendicoliths when performing an appendicectomy for perforated appendicitis. As with any retained endogenous or exogenous material, the potential for early and delayed complications is not insignificant. The patient underwent 4 attempts at percutaneous drainage, each leading to the recurrence of the abscess because even though the appendicolith was detected at the first CT scan 3 weeks after the appendicectomy, no effort was made to remove it. As with any dropped organic material or foreign body involved with an infective process, it is essential to extract it if further infections are to be prevented.

Like spilled gallstones [10], dropped appendicoliths can cause a plethora of intra- and extra-abdominal complications. Spillage of the appendicolith usually occurs in the pelvis, paracaecal region, Morison's pouch (posterior subhepatic space), or between loops of the small bowel [1,^2^,11]. However, the appendicolith can migrate to any part of the peritoneal cavity, the muscles of the abdominal wall, and even transdiaphragmatically [1,^2^,12]. This migration can result in complications such as intra-abdominal abscesses [7], hepatic abscesses [13], intramuscular abscesses [5], fistulae [1], pneumonia with empyema [12], migration to the subcutaneous tissues and delayed wound healing [1,14].

Perihepatic abscesses resulting from dropped appendicoliths are uncommon and a review of the literature has revealed 11 cases in 10 reports, including the present one (Table 1) [[6], [7], [8], [9],^11^,[15], [16], [17], [18]]. All 11 patients had appendicectomy for perforated appendicitis and presumably, the dropped appendicoliths were secondary to the perforation. The age of the patients ranged from 6 to 37 years and they presented with signs of inflammation or sepsis between 7 days and 2 years after appendicectomy, and were diagnosed to have perihepatic abscesses with dropped appendicoliths on CT or ultrasound scan, with CT being the best diagnostic modality. One patient was successfully managed with antibiotics without drainage of the abscess [17], but this is the exception rather than the rule. Two patients had successful percutaneous drainage of the abscess with subsequent (up to several weeks later) percutaneous extraction of the appendicolith [7,18]. One patient had successful percutaneous drainage of the abscess without retrieval of the appendicolith [11] and two had percutaneous drainage of the abscess with subsequent surgical retrieval of the appendicolith [11,15]. Three patients had laparoscopic drainage of the abscess with retrieval of the appendicolith [6,^8^,16], one of whom had unsuccessful ultrasound-guided drainage [16], and another needed a second laparoscopic procedure to remove the second of two appendicoliths [8]. Finally, two patients (including the present one) underwent laparotomy with drainage of the abscess and retrieval of the appendicolith [9]. It is important to retrieve the appendicolith as drainage of the abscess alone usually leads to persistent or recurrent abscess formation, as in the present case and others [8,11].

**Table 1:** Perihepatic abscesses due to dropped appendicoliths.

Author and year	Country	Presentation	Treatment
Lossef, 2005 [15]	USA	An 11-y-old female presented with intermittent fevers over a 4-mo period after laparoscopic appendicectomy for a ruptured appendix. CT showed an 8 cm abscess in the Morison's pouch containing a 3-4 mm focal calcification believed to be a retained appendicolith.	CT-guided percutaneous drainage of abscess done initially. Follow-up CT showed complete resolution of the subhepatic abscess but persistence of a small calcification between the liver and right kidney. Appendicolith removed surgically via a posterior right intercostal approach after CT-guided needle localization.
Singh, et al., 2008 [11]	USA	Two cases of dropped appendicoliths and abscess formation in Morison's pouch (34-y-old female 1 wk after appendicectomy, details for the other patient not mentioned). Both diagnosed on CT.	One patient had successful percutaneous catheter drainage of the abscess. In the other, the Morison's pouch abscess had a large phlegmonous component that prevented adequate catheter drainage and needed surgical drainage.
Maatouk, et al., 2011 [16]	UK	A 17-y-old female presented a year after laparoscopic appendicectomy for perforated appendicitis with intermittent right upper quadrant pain, nausea and vomiting. CT revealed a 4 × 3 cm abscess posterolateral to segment VI of the liver with a central 15 mm high-density lesion, suggestive of a retained appendicolith.	Ultrasound-guided drainage was attempted but failed secondary to an iatrogenic pneumothorax and inability to localize the lesion. Underwent laparoscopic drainage of the abscess and retrieval of the appendicolith.
Whalley, et al., 2013 [6]	UK	A 21-y-old male presented with a 3-d history of shortness of breath, productive cough, fatigue, fever, and night sweats, associated with right upper quadrant pain 4 mo after laparoscopic appendicectomy for perforated appendicitis. CT showed a right subphrenic abscess indenting the right lobe of the liver and containing a calcified foreign body suggestive of a retained appendicolith was made.	Laparoscopic drainage of the abscess and retrieval of the appendicolith.
Black, et al., 2013 [17]	USA	A 6-y-old female underwent laparoscopic appendicectomy for acute appendicitis with intraluminal appendicoliths on ultrasound. Presented 2 wk later being unwell. An ultrasound scan revealed a 3.2 × 2.6 × 1.7 cm hypoechoic lesion with internal calcifications, suggesting a perihepatic space abscess.	Treated with antibiotics. Serial ultrasound scans showed a decrease in size of the abscess.
Hovis and Jeffrey, 2017 [18]	USA	CT scan of a 24-y-old man scan showed an enlarged and inflamed appendix with 2 faecoliths and a perforation. He presented with right upper quadrant abdominal pain and fever 3 mo after a laparoscopic appendicectomy. Ultrasound and CT revealed a complex perihepatic fluid collection and a 10 mm calcification consistent with an appendicolith adjacent to segment V of the liver.	The abscess was drained percutaneously and after subsequent serial dilation of the tract, the appendicolith was successfully retrieved with a Dormia basket.
Singh, et al., 2019 [8]	USA	Laparoscopic appendicectomy for gangrenous perforated appendicitis in a 10-y-old male. Two appendicoliths seen of preoperative CT not retrieved. On postoperative day 18 revealed 2 small complex fluid collections measuring approximately in the periphery of the posterior right hepatic lobe with surrounding inflammation with calcified foci within them.	Laparoscopic drainage of the abscesses was successful but only one of the two appendicoliths was removed. Postoperative CT confirmed the presence of the retained appendicolith which was removed after an additional ultrasound-guided laparoscopic surgery.
Abdullah, et al., 2019 [7]	USA	Recurrent right subdiaphragmatic abscess 2 and 7 y after appendicectomy for perforated appendicitis in a 24-y-old male.	CT guided percutaneous drainage of abscess with serial upsizing of drain over several weeks followed by percutaneous endoscopic retrieval of appendicolith.
Bašković, et al., 2022 [9]	Croatia	Multiple intra-abdominal abscesses (right subhepatic, pericaecal, and retrovesical) with appendicolith in subhepatic abscess in an 8-y-old male 11 d after open appendicectomy for gangrenous perforated appendicitis.	Laparotomy, drainage of abscesses, and retrieval of appendicolith.
Present case	UK	Recurrent retrohepatic abscess in a 37-y-old woman after appendicectomy for perforated appendicitis. 4 × percutaneous drainage between 3 wk and 7 y after appendicectomy without removal of appendicolith.	Laparotomy, drainage of retrohepatic abscess and retrieval of appendicolith.

In the event of performing an appendicectomy for perforated appendicitis, surgeons should make an effort to identify and retrieve dropped appendicoliths as this may prevent subsequent abscess formation and other complications. This is particularly important if the patient has been shown to have dropped appendicoliths on preoperative imaging. Dropped appendicoliths can be visualized on CT scan as a high attenuation zone [13]. Ultrasound is particularly beneficial for paediatric patients due to lack of exposure to radiation, lower costs, and reduced invasiveness.

## Conclusion

Although a rare postappendicectomy complication, there should be a high clinical suspicion for a dropped appendicolith in patients with intra-abdominal abscesses and a previous history of an appendicectomy. Late identification can result in recurrence of abscesses and may even put patients at risk of sepsis, empyema, and peritonitis. Treatment of abscesses secondary to dropped appendicoliths may be percutaneous, laparoscopic, or via conventional open surgery, but it is important to retrieve the appendicolith if recurrent abscess formation is to be avoided. Awareness of the possibility of dropped appendicoliths is vital for both radiologists and surgeons to prevent further complications and recurrent hospital admissions for patients.

## Author contributions

Study design: All authors. Data collection: All authors. Data interpretation: All authors. Writing of the manuscript: SS, MK, DNL

Critical review of the manuscript: All authors. Final approval: All authors. All authors had access to the data.

## Data sharing

No additional data to share.

## Patient consent

The patients have provided written informed consent for this submission. We can provide the journal a copy if needed.
